# Atlanta Academy of Medicine

**Published:** 1877-03

**Authors:** J. S. Todd

**Affiliations:** Reporter


### ATLANTA ACADEMY OF MEDICINE.

J. S. TODD, M.D., REPORTER.

ATLANTA, January 30, 1877.

The President, V. H. Taliaferro, in the chair.

Dr. Calhoun reported an unique case of accident to the eye, resulting in better vision than patient had ever enjoyed before.

The case in question was a double dislocation of lens. The patient, a man aged 40, in robust health, while violently exerting himself to lift his wagon out of the mire, caused the dislocation in one eye. He did not, however, recognize the accident immediately. His wife said the eye looked as though there was a drop of oil in it. Soon he had general inflammation of eye, which resisted treatment by general practitioner for six weeks. Dr. C. found the crystiline lens partially opaque, and in anterior chamber of eye, in front of the iris, the eye still much inflamed and tender. Under chloroform, he performed the cataract operation, at the junction of the cornea and sclerotic, without an iridectomy, extracting the lens. There was a rapid subsidence of pain and inflammation, followed by speedy recovery. Leaving here for Montgomery, Alabama, in getting on cars he exerted himself considerably, and also fell. By the time he reached that place the other eye was inflamed, etc. The same appearance was present in the eye when he returned as was observed in opposite eye when first seen by him. Same operation, with like result, in that eye. When he left the last time, after suitable glasses had been procured, he declared he never before knew how blind he had been all his life. Dr. C. thought that both ligaments of lens were ruptured by first accident, but that it was less complete in last, and required but slight violence to complete it, which was furnished by fall in car, etc.

Dr. Ford reported a case of hydrophobia, fatal on third day. The boy—a negro—was bitten on the bare leg in November last, by an animal that was supposed to be mad. The symptoms were tonic convulsions of the muscles of deglutition, and slight drawing of the head backwards; unable to eat solid or liquid food. Attempts to swallow brought on the spasms; touching the cicatrix also occasioned them. They were controlled by morphine, if no effort at deglutition was attempted. Dr. Miller, who saw the patient with him, although he had never seen a case of rabies, gave it as his opinion that the symptoms were characteristic of that affection as it was described in the books.

Dr. Miller also said that he did not believe that dogs were any more apt to become affected during one season than another. The period of incubation was uncertain. All persons bitten by same animal were not invariably affected with hydrophobia; on the other hand, but a few are so affected. The animal diseased may, and does, sometimes recover, and the person or animal bitten by him die.

Dr. Wilson said that other children had been bitten through the clothing, and not affected.

Dr. J. M. Boring, last Wednesday night, attended a lady in a short, easy, natural labor, that brought forth a fine, well developed boy, weighing nine and a half or ten pounds. Tied the cord with a tape string, and with his usual care—and he is always very careful to have the knots firm and secure, having had hemorrhage to occur. Called at nine A.M. next day, and found the bandage and clothing saturated with blood. Upon removing the dressing, he found the ligature intact, but applied another one half an inch lower down the cord. The cord, which was large and healthy in all respects when the child was first born, was now black and flaccid. That afternoon he applied another ligature lower, as bleeding was still going on. At night, the bleeding continuing, he cleared away the stains and blood, and discovered that at the abdomen blood was oozing. He continued to ligate as long as any cord was left to tie, and applied astringents, without arresting the hemorrhage. The child, he was told, vomited blood, and he verified this supposition by an inspection of the emesis on the clothing. It died on Saturday, the bleeding being the immediate cause of death. After death he noticed splotches on its body. The blood that came from it did not coagulate, even in contact with the astringents. Never met but one case like it, but in that case he succeeded in saving the child, but it was never healthy or of sound mind, and died young. On Friday the child became jaundiced, and passed from its bowels dark, grumous looking material, which may have been blood. His diagnosis was purpura hemorrhagica. The parents were not scrofulous or syphilitic. Hemorrhage from the naval, at its insertion into the belly, does occur five, six, or seven days after delivery, or whenever the cord sloughs; but this bled from the cord itself, or oozed bodily through it, more correctly speaking.

Dr. Miller said that a well marked case of purpura hemorrhagica in a child was rare. When there is bleeding from the cord after sloughing has taken place, death was the rule. Astringents, compresses, etc., all fail. If we could ligate the artery and not wound the peritoneum, he would advise it unhesitatingly. Had saved one case by transfixing the naval with needles and making figure of eight knot over them; but even in that case the needles sloughed out and left on him a smooth scar, which is apparent still, though the patient is now a man. When children become jaundiced, the emesis and catharsis often assume such a dark color that they are apt to be mistaken for blood. The splotches spoken of by Dr. Boring were post-mortem, the result of gravity, doubtless. The vomiting of blood in purpura is rare. He was sorry to disagree with Dr. B., but did not think it purpura. Children are often born with the cord defective, and as a rule they die

The President rather agreed with Dr. Boring until he heard the testimony of Dr. Miller. He then said he was not satisfied in his own mind what the cause of bleeding was. Remarked that syphilis was often a factor when we least expected it; and when parents denied ever having had it.

Dr. J. S. Todd called the attention of members to a very stubborn, and to him rare, case of hyperesthesia of the skin. He asked for a remedy which would relieve. The patient, a civil engineer, æt. 30, in tolerable good health, described his sufferings as intense, and occurring whenever, from physical exertion, or going too near the fire when the surface was chilled, he thereby suddenly elevated the temperature. The slightest sudden elevation of temperature, from any cause, gave him a burning, pricking pain all over his body, that could only be compared to the tortures of the damned. His bowels were regular, appetite tolerable, urine large in quanity and frequently voided. He had been similarly affected several winters ago. During the summer, when the pores of the skin are open, he does not have the spells. During cold weather he does not perspire sensibly. An examination of the urine for sugar and albumen, while it revealed the absence of both these substances, showed a large preponderance of urea; so large that nitric acid and two-thirds urine, of specific gravity 128, yielded such an abundance of crystals that their bulk covered almost as much space in the test tube as was occupied by urine and acid. This condition of urine had been relieved, but still the patient has an occasional recurrence of hyperesthesia. Alkaline diuretics and citric acid drinks corrected the urinary trouble. He put the patient on a pill of arsenic, ex. aconite, and quinine. The aconite was given for its action in benumbing cutaneous sensibility.

Dr. Miller thought the whole trouble lay in suppression of the cutaneous secretion. A good sweat, followed by repeated small doses of antimony, was indicated. People in the country, a long time ago, used to boil ears of Indian corn, and while they were hot and steaming, placed them under the blankets with patient, giving them thereby, in effect, a capital vapor bath. The excess of deposit in urine was occasioned by the vicarious office the kidneys had to perform for the skin.

Academy adjourned.

Note.—Dr. Todd acted on the suggestion of Dr. Miller, with the happiest results. Up to this writing the patient has had but one slight spell since he took the first Turkish bath. He has taken but two, but can now exercise and go near a fire with impunity. The only effect of over-exertion or caloric is free perspiration, unattended by disagreeable sensations.—*Reporter.*

*February 24th.*

---

Atlanta, Feb. 6, 1877.

The Vice-President, Dr. John M. Boring, presiding.

Dr. J. T. Johnson presented a calculus, of irregular shape, a little larger than a pea, which he extracted from the urethra. It occurred in a patient of his whom he had treated for stricture. The urethra had been dilated so that it would admit of the passage of the largest sound. While treating him, and for some time before, he had brick-dust deposit in the urine. He doubts not but had the urethra been of small calibre, it would have—remaining in the bladder—acted as a nucleus for stone, which would have required lithotomy to remove it. As it was, the calculus lodged one and a half inches from meatus. The operation of extracting was performed with forceps; the precaution, of course, being taken to grasp the penis behind the calculus, so that it would not slip back. He thinks in all cases where there is phosphatic or uric acid diathesis or sediment in the urine of inorganic matter, that it would be well to dilate the urethra to its full capacity, so as to admit of the escape of small accumulations. Dr. Johnson also exhibited a stone extracted by ordinary lateral operation—phosphatic in character. It was very brittle, crumbling easily under the pressure of fingers.

Dr. Todd asked if he did not think that it was a fine case for lithotrity; and also asked his opinion of that operation.

Dr. Johnson's opinion of lithotrity was not good. In the old and feeble it would not do at all, on account of vesical and urethral irritations. In the young and robust lithotomy was more satisfactory, less dangerous, and the cures were more rapid.

Dr. Johnson also exhibited a calculus extracted from a female, by Dr. Banks, of Griffin. The concretion was triangular in shape, with the angles so rounded off that it bore a very striking resemblance to the human heart. It was one and three-quarter inches long, one and a half wide at its base, and three-quarters thick. It was extracted entire through the urethra, without rupturing or cutting that canal; the woman being under chloroform. Dr. B. first introduced an olive-pointed sound of large calibre into the urethra, which was followed by successively larger ones, up to No. 20 English, or the largest size; then alternately his little finger, fore-finger and thumb; when forceps were applied and the stone slowly extracted. It was not followed by incontinence of urine, even of a temporary nature. Stated further that he might have cut the urethra with impunity, toward the vagina, but upwards towards symphesis pubis preferably. The dilatation and extraction was all done at one sitting.

Dr. Johnson also exhibited a biliary calculus, found in the peritoneal cavity, by Dr. Nutting, in a subject at the dissecting room of the College. There were numerous abscesses in liver. Not knowing the previous history of case, of course all that might be said was hypothetical. The stone was smooth, and about the size of a small chestnut.

Academy adjourned.

---

ATLANTA, February 13, 1877.

Vice-President J. M. Boring, in the chair.

Dr. J. G. Westmoreland called attention to a case of a patient of his who had been sick for five weeks. At first eyes were red, showing great congestion of the conjunctiva—pain in the head, over eyes. A blister to occiput dissipated this pain, also that in the back of the head—the tongue has been red continually, and there has been occasional diarrhœa. The patient had been sick the first two weeks with an attack of sub-acute hepatitis. Has had evening exacerbations, pulse generally from 80 to 110. No thoracic trouble. The erect position causes so much pain in the head that the patient is still unable to sit up. He does uot think it a case of typhoid fever on account of the time it has lasted and the freedom occasionally enjoyed from fever in the morning. He had used quinine without effect.

Dr. Todd asked if he used a clinical thermometer.

Dr. Westmoreland, no.

Dr. Todd thought the case one of typhoid fever, and said there was no one of our senses so liable to mislead as that of touch, and mentioned a case on which he took notes where frequently there was a moist, cool skin, pulse 66 to 70, while the thermometer recorded as high as 104°.

Dr. Westmoreland said that when the circulation was controlled by varatrum, such mistakes were liable to occur, and it was not always that quickness of pulse is present in fever—as the brain is often so pressed that the pulse is slow from that cause.

Dr. Todd asked if there was tenderness in the right iliac fossa.

Dr. Westmoreland, did not examine for it.

Dr. Todd then asked if there was gurgling.

Dr. Westmoreland did not know, and did not care.

Dr. Todd then asked if he did not think that Pyer's glands were involved in typhoid fever.

Dr. Westmoreland said he did not think they had anything to do with it, except incidentally.

Dr. Todd expressed surprise at Dr. W.'s position. In his practice it had been the exceptional and uncomplicated case which recovered in three weeks. For it to last five weeks was by no means rare.

Dr. Boring : While he had seen no typhoid fever, thought Dr. Westmoreland's case one.

Dr. Taliaferro had noticed an eruptive fever resembling roseola, complicated, occasionally, with pharyngitis and tonsilitis. Is it a precursor of scarlatina?

Dr. J. G. Westmoreland said it impressed him as following epidemics of measels rather than going before scarlet fever.

Dr. Boring had not seen scarlet fever since December.

Dr. Taliaferro prescribed belladonna and bromide of potash with little faith in them, and without benefit, to a boy eight years of age, who had suffered for a number of years with incontinence of urine. A sound revealed an exceedingly irritable urethra and a stricture at the bulb. There was also slight adhesion of prepuce to the gland. The stricture was organic. After repeated attempts, extending over several days, he succeeded in introducing a No. 5, English sound. This had been introduced several times, and the boy could now go for four or five days after the introduction without wetting the bed.

Dr. J. T. Johnson: A stricture of the meatus often led to incontinence of urine and gave rise to the deeper strictures, both organic and spasmodic.

Dr. Westmoreland said that strictures were sometimes congenital, and instanced a case in question. Thought in this case there were grounds for such a deduction.

Dr. McIntosh agreed with Dr. Westmoreland, and said, a congenital stricture at the bulb was as reasonable of belief and as apt to occur as one at the meatus. A contracted prepuce, or one partially adherent, by retaining the secretion impairs the health. The reflex irritation caused by inflammation of the gland had caused paralysis and epilepsy.

Dr. Sayre, of New York, had written a paper on the subject, and had cured a patient of paralysis by circumcision. He therefore thought the stricture not the only factor in causing the incontinence, and hence advised that circumcision should also be practiced. Had himself seen the general health impaired, paralysis and incontinence ensue, from adherent prepuce.

Dr. Taliaferro thanked Dr. McIntosh for the suggestion, and said he would act on it. Had already advised circumcision.

Dr. Westmoreland : The irritability of the bladder may have caused the itching in gland, and constant pulling at it gave rise to the hypertrophy of it alluded to by Dr. Taliaferro; relief of lower stricture would be all that was necessary in his opinion.

Dr. McIntosh : It was not essential that there should be inflammation of the gland or prepuce to cause these reflex phenomena, for the tenderness at point of adhesion, which always exists more or less, is sufficient to cause them. His observation led him to believe that boys frequently pulled at the prepuce where it was long or adherent.

Academy adjourned.

---

Atlanta, February 20, 1877.

The President, Dr. V. H. Taliaferro, in the chair.

Dr. Armstrong was called to see a patient, forty years of age, who, upon examination, appeared to be suffering from an acute inflammation of the submaxillary gland. Physical examination with finger shows that Wharton's duct was enlarged and inflamed. The next day, upon pressing upon the duct, there oozed a whitish, milky fluid, one-half an inch in rear of the orifice of the duct. For a year there had been some tenderness in and about the gland. He suspected a calculus, and accordingly made an incision along the course of the duct. No pus escaped, the tissues were very hard, he found nothing. Next day his wife handed him a calculus which he exhibited to the Academy. It was about the size and shape of an orange seed, and his wife stated that she observed something sticking in the wound, and wiping it out, the calculus came away. It was phosphatic in character.

Dr. H. L. Wilson was called on Thursday to see a gentleman aged forty-five, who had previously enjoyed perfect health, he being a temperate man of regular habits. He complained of severe frontal headache, which came on suddenly after breakfast. He prescribed ½ gr. of morphia and 8 grs. quinine. Next day still suffering with acute lancinating pain in head, and the conjunctiva was much congested. Suspecting arachinitis, he ordered a blister to the spine, and that night 20 grs. calomel. Saturday worse and unconcious; pupils dilated; pulse 130. Cupped him profusely over the temple and gave 40 grs. calomel. He died at four that eveniong without an operation on the bowels. He did not notice that there was any opisthotonos; if present it was slight. There was some evidence of paralysis; he was unable to move or help himself at all after Friday.

Dr. Baird thought it was cerebral meningitis on account of the severity of the pain and its extension. Spoke of the bromide of potassa and fluid extract ergot as remedies for congestion of the brain and headaches, dependant on congestion, in the highest terms of commendation. Said the remedies must be pushed. Small doses of either will disappoint you. Mentioned a case recently under his care, of a lady who had all the symptoms of violent congestion of the brain relieved by potas. bro., ℥ss.; fld. ex. ergot, ℥j.; given at a dose every two hours for twenty-four, which was lessened to three times a day, the dose of bromide being increased to a drachm. It was followed by prompt relief of the symptoms of congestion, and occasioned bromidism, viz: weakness, physical and mental; leeches were also applied, and two oz. of blood taken. After bromidism, left off bromide and gave the ergot in drachm doses pro re nata. The symptoms of congestion were marked, the patient had illusions, hallucinations, etc. She was confined to her bed from weakness for three weeks.

Dr. Baird thought it acted by causing a contraction of the muscular coats of the arteries and arterioles. It has no effect upon the healthy non-pregnant uterus. He would give them in meningitis.

Dr. Todd thought that the length of time Dr. Baird's patient was sick spoke very little for the efficacy of the medicine employed. A blister to the spine, cold to the head, and a brisk mercurial purge would have been better treatment. He attributes some of the mental symptoms to the large doses of ergot.

Dr. Baird said cold was used to the head, and as the patient already had diarrhœa he did not think any active purgative indicated. If any cathartic at all was admissible, aloes was the one he would have used, and not calomel.

Dr. Taliaferro agreed with Dr. Baird, as to the propriety of giving ergot and bro. potass. in congestion of the brain and congestive headaches. Ergot in the non-pregnant uterus has no appreciable effect unless it was congested, hypertrophied or there is a fibroid of the uterus, when it will cause contraction. Is satisfied that Prof. Byford, of Chicago, is correct in saying that ergot will bring about disintegration and expulsion of fibroid tumors of the uterus. Is also convinced that Dr. Johnson, (J. M.) is correct in according to it aphrodisiac properties.

Dr. Taliaferro also reported a case of diphtheria, but as he had seen but little of the disease, detailed some of the most prominent symptoms, asking to be corrected in his diagnosis if any member thought the evidence not sufficient to substantiate a case. The child is eight years of age, tonsils enlarged and covered with white patches which he cannot scrape off; a brother of child also has a membrane of same character on one tonsil. Sat. sol. chlorate potass., quinine and turpentine are the remedies employed.

Dr. H. L. Wilson has a case of skin disease, for which he gave Fowler's Solution, beginning with two drops, and directed a gradual increase every second day. On taking three drops the patient complained of pain in the eyes and a feeling of tightness arcoss the forehead. There was genuine puffiness about the lids. He asked if he might expect good from so small a dose; would it have its specific effect?

Dr. Armstrong thought it an idiosyncrasy.

Dr. Taliaferro finds many persons who cannot bear more than three drops. Where puffiness headache, etc., are produced should expect it to do the same good as when they were deferred for larger doses.

